# Cytotoxic and Radical Scavenging Nor-Dammarane Triterpenoids from *Viburnum mongolicum*

**DOI:** 10.3390/molecules18021405

**Published:** 2013-01-24

**Authors:** Xiaohua Wang, Wei Wang

**Affiliations:** 1 Department of Pharmacy, No.202 Hospital of PLA, Shenyang 110003, China; 2 Department of Pharmacy, No.455 Hospital of PLA, Shanghai 200052, China

**Keywords:** *Viburnum mongolicum*, Caprifliaceae, nor-dammarane triterpenoids, cytotoxicity, radical scavenging activities

## Abstract

The ethanol extract of the whole plants of *Viburnum mongolicum* afforded six new nor-dammarane triterpenoids: 3*β,*12*β*-dihydroxy-25,26,27-trinordammara-22-en-24,20-olide (**1**), 3*β,*12*β*-dihydroxy-24*α*-methoxy-25,26,27-trinordammara-20,24-epoxy (**2**), 3*β*-*O*-acetyl-12*β*-hydroxy-23,24,25,26,27-hexanordammarane-20-one (**3**), 12*β*-*O*-acetyl-15*α*-hydroxy-17*β*-methoxy-3-oxo-20,21,22-23,24,25,26,27-octanordammanrane (**4**), 12*β*-*O*-acetyl-15*α*,17*β*-dihydroxy-3-oxo-20,21,22-23,24,25,26,27-octanordammanrane (**5**), and 12*β,*15*α*-dihydroxy-3-oxo-17-en-20,21,22-23,24,25,26,27-octanordammanrane (**6**), together with two known nor-dammarane triterpenoids, 12*β*-hydroxy-3-oxo-24*α*-methoxy-25,26,27-trinordammara-20,24-epoxy (**7**) and 3*β,*12*β*-dihydroxy-23,24,25,26,27-hexanordammarane-20-one (**8**). The structures of the isolated compounds were established based on 1D and 2D (^1^H-^1^H COSY, HMQC, and HMBC) NMR spectroscopy, in addition to high resolution mass spectrometry. The isolated compounds were tested *in vitro* for cytotoxic potential against seven tumor cell lines and radical scavenging activities. Compounds **4**–**6** exhibited significant cytotoxic activities against all tested tumor cell lines and radical scavenging activities against ABTS^·+^ radicals comparable with the standard drug Trolox.

## 1. Introduction

**Figure 1:**
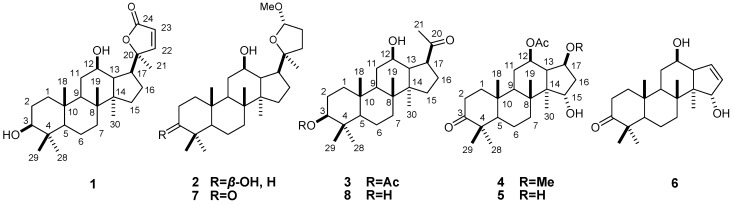
Structures of compounds **1**–**8**.

The genus *Viburnum*, which belongs to the family of Caprifliaceae, comprises more than 230 species [1,2]. The majority of them are endemic and mainly distributed in the temperate or subtropical zones, particularly from South America (Peru) to South-East Asia (Philippines and Malaysia) [3,4]. Among them, 80 species naturally occur in China [5]. *Viburnum* species have diuretic, antispasmodic and sedative properties, mainly for uterine excitability and are commonly used as a traditional Chinese herbal medicine as an astringent, sedative, emmengagogue and the treatment of other health problems [6,7]. The genus *Viburnum* is known to be rich in iridoids, iridoid glycosides, sesquiterpenes, vibsane diterpenes, triterpenes, triterpene saponins, flavonoids, lignans, coumarins, and caffeoyl acid derivatives [8,9,10,11,12,13,14,15,16,17,18,19,20,21,22,23,24]. As part of our search for novel and bioactive constituents, *Viburnum mongolicum* (Pall.) Rehd., an evergreen shrub, has been investigated. It is widely distributed in the Menggu, Hebei, Shanxi, Ningxia, Gansu and Qinghai provinces of China. A literature search revealed that phytochemical and pharmacological studies have rarely been undertaken within this species [5]. As part of our search for novel and bioactive constituents, a phytochemical study of the chloroform-soluble fraction of the 80% EtOH extract of *V. mongolicum* was undertaken, whichfurnished six new nor-dammarane triterpenoids, 3*β,*12*β*-dihydroxy-25,26,27-trinordammara-22-en- 24,20-olide (**1**), 3*β,*12*β*-dihydroxy-24*α*-methoxy-25,26,27-trinordammara-20,24-epoxy (**2**), 3*β*-*O*- acetyl-12*β*-hydroxy-23,24,25,26,27-hexanordammarane-20-one (**3**), 12*β*-*O*-acetyl-15*α*-hydoxy-17*β*- methoxy-3-oxo-20,21,22-23,24,25,26,27-octanordammanrane (**4**), 12*β*-*O*-acetyl-15*α*,17*β*-dihydoxy-3- oxo-20,21,22-23,24,25,26,27-octanordammanrane (**5**), and 12*β,*15*α*-dihydoxy-3-oxo-17-en-20,21,22-23,24,25,26,27-octanordammanrane (**6**), and two known compounds, 12*β*-hydroxy-3-oxo-24*α*-methoxy-25,26,27-trinordammara-20,24-epoxy (**7**) and 3*β,*12*β*-dihydroxy-23,24,25,26,27-hexa- nordammarane-20-one (**8**) (Figure 1). The six new compounds are endowed with three different triterpenoid skeletons: **1** and **2** are trinordammaranes, **3** is a hexanordammarane, and **4**–**6** are octanordammaranes. This paper describes the isolation and structure elucidation of the new compounds, as well as the tested *in vitro* cytotoxic and radical scavenging activities of compounds **1**–**8**.

## 2. Results and Discussion

### 2.1. Chemistry

**Figure 2:**
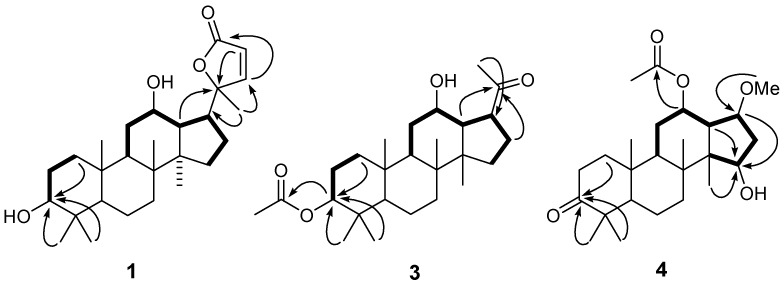
Key HMBC (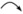) and ^1^H-^1^H COSY (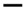) correlations of compounds **1**, **3** and **4**.

Compound **1** was obtained as a white amorphous powder. Its positive HR-ESI-MS spectrum showed a quasimolecular ion peak at *m/z* 453.2984 [M+Na]^+^, consistent with the molecular formula C_27_H_42_O_4_ (calcd. for C_27_H_42_O_4_Na 453.2981), accounting for seven degrees of unsaturation. The IR spectrum indicated the presence of OH (3450 cm^−1^) and C=O (1702 cm^−1^) groups, as well as a C=C bond (1640 cm^−1^). ^1^H-NMR signals for six methyl groups [*δ*_H_ 1.45, 0.99, 0.91, 0.89, 0.84 and 0.79 (each, s)] and for two oxygenated CH groups [*δ*_H_ 3.21 (H-3) and 3.62 (H-12)] were observed (Table 1). The characteristic^ 13^C-NMR spectrum, which was similar to that of the known compound cylindrictone B [25], indicated the 27 skeleton carbons in **1** as composed of six methyls, seven methylenes, eight methines (two oxygenated and two olefinic), five quaternary carbons and one C=O group (*δ*_C_ 172.1). The distinct difference was that the ketone carbon signal at C-3 in cylindrictone B was replaced by an oxygenated methine (*δ*_H_ 3.21; *δ*_C_ 78.5) in **1**, which was supported by the observation of a CH_2_-CH_2_-CH**-**O spin system in the ^1^H,^1^H-COSY spectrum and the HMBC correlations between C-3 (*δ*_C_ 78.5) with H-1 (*δ*_H_ 1.03 and 1.69), H-5 (*δ*_H_ 0.78), H-28 (*δ*_H_ 0.79) and H-29 (*δ*_H_ 0.99) (Figure 2). Analysis of the HMBC spectrum of **1** assigned the OH group to C-12 due to the long-range correlations of H-9 and H-17 to C-12, and of H-12 to C-14. The C=C bond was located between C-22 and C-23 because of the presence of HMBC correlations from H-22 and H-23 to C-24 and C-20 respectively. The relative configuration of **1** was established through an analysis of the ROESY data. The *β*-orientation of the 3- and 12-OH was deduced from the correlations of H-3 with H-1ax and H-5, and of H-12 with H-9 and H-30. The presence of ROESY correlations of H-21 with H-17 and H-12 verified the *α*-orientation of Me at C-21. Thus, **1** was identified as 3*β*,12*β*-dihydroxy-25,26,27-trinordammara-22-en-24,20-olide.

Compound **2** was obtained as a white amorphous powder. Its positive HR-ESI-MS spectrum showed a quasimolecular ion peak at *m/z* 471.3454 [M+Na]^+^, consistent with the molecular formula C_28_H_48_O_4_ (calcd. for C_28_H_48_O_4_Na 471.3450), accounting for five degrees of unsaturation. The IR spectrum absorptions at 3425 cm^−1^ indicated the presence of the OH group. The ^1^H-NMR spectrum (Table 1) and ^13^C-NMR data (Table 2), along with the DEPT and HSQC experiments, showed that the carbons were seven methyls (including one methoxy group), nine methylenes, seven methines (including three oxygenated methine groups), and five quaternary C-atoms (including one oxygenated C-atom). These NMR data were quite similar to those of 12*β*-hydroxy-3-oxo-24*α*-methoxy-25,26,27-trinordammara-20,24-epoxy (**7**), except for the replacement of the ketone carbonyl of C-3 by a hydroxymethylene moiety (*δ*_H_ 3.17; *δ*_C_ 78.4). This was supported by HMBC correlations from the H-3 to C-1 (*δ*_C_ 38.8), C-5 (*δ*_C_ 56.0), C-28 (*δ*_C_ 15.2), and C-29 (*δ*_C_ 27.9). These HMBC correlations of the oxygenated H-24 (*δ*_H_ 4.93) with the oxygenated quaternary C-atom C-20 (*δ*_C_ 80.4) and methoxy (*δ*_C_ 55.4) suggested that C-20 and H-24 were linked via O bridge to form a lactol ring, and the methoxy group was placed at C-24. The relative configuration of **2** was confirmed by a ROESY experiment. In the ROESY spectrum, the correlations of H-3/H-1ax and H-5 indicated that the OH-3 was *β*-oriented. The *α*-orientation of the methoxy group at C-24 was determined to be identical to that of **7** on the basis of ROESY correlation of H-24 with Hax-22 and of H-21 with Heq-22. Thus, the structure of **2** was established as 3*β,*12*β*-dihydroxy-24*α*-methoxy-25,26,27-trinordammara-20,24-epoxy.

**Table 1:** ^1^H-NMR data of compounds **1**–**6** in CDCl_3_ (*δ* in ppm and *J* in Hz).

No.	1	2	3	4	5	6
1ax	1.03 (*ddd*, 13.8, 13.3, 3.6)	1.00 (*ddd*, 14.0, 13.5, 3.6)	1.02 (*ddd*, 14.0, 13.2, 3.6)	1.47 (*ddd*, 14.0, 13.3, 3.6)	1.44 (*ddd*, 13.8, 13.6, 3.6)	1.45 (*ddd*, 13.8, 13.6, 3.6)
1eq	1.69 (*ddd*, 13.3, 4.0, 3.6)	1.66 (*ddd*, 13.5, 4.0, 3.6)	1.68 (*ddd*, 13.2, 4.0, 3.6)	1.95 (*ddd*, 13.3, 4.0, 3.6)	1.94 (*ddd*, 13.3, 4.0, 3.6)	1.93 (*ddd*, 13.5,3.8, 3.6)
2ax	1.59 (*m*)	1.56 (*m*)	1.59 (*m*)	2.38 (*ddd*, 14.0, 13.3, 3.6)	2.36 (*ddd*, 13.8, 13.6, 3.6)	2.39 (*ddd*, 13.8, 13.6, 3.6)
2eq	1.67 (*m*)	1.64 (*m*)	1.66 (*m*)	2.52 (*ddd*, 13.3, 4.0, 3.6)	2.48 (*ddd*, 13.3, 4.0, 3.6)	2.47 (*ddd*, 13.5, 3.8, 3.6)
3	3.21 (*dd*, 14.0, 4.0)	3.17 (*dd*, 14.0, 4.0)	4.39 (*dd*, 14.0, 4.0)	-	-	-
5	0.78 (*dd*, 14.0, 3.8)	0.76 (*dd*, 13.8, 3.8)	0.79 (*dd*, 13.8, 3.6)	1.37 (*dd*, 13.8, 3.8)	1.35 (*dd*, 13.5, 3.0)	1.36 (*dd*, 13.0, 3.0)
6ax	1.47 (*m*)	1.46 (*m*)	1.48 (*m*)	1.44 (*m*)	1.42 (*m*)	1.48 (*m*)
6eq	1.62 (*m*)	1.58 (*m*)	1.60 (*m*)	1.58 (*m*)	1.56 (*m*)	1.59 (*m*)
7ax	1.58 (*ddd*, 14.0, 13.5, 3.8)	1.55 (*ddd*, 14.0, 13.3, 3.8)	1.57 (*ddd*, 14.0, 13.5, 3.8)	1.54 (*ddd*, 13.8, 13.3, 3.8)	1.53 (*m*)	1.54 (*m*)
7eq	2.18 (*ddd*, 13.5, 4.0, 3.6)	2.15 (*ddd*, 13.3, 4.0, 3.6)	2.17 (*ddd*, 13.5, 4.0, 3.6)	2.20 (*m*)	2.18 (*m*)	2.19 (*m*)
9	1.55 (*dd*, 13.6, 3.0)	1.53 (*dd*, 13.6, 3.2)	1.55 (*dd*, 13.6, 3.2)	1.51 (*dd*, 13.0, 3.0)	1.49 (*dd*, 13.8, 3.0)	1.52 (*dd*, 13.5, 3.0)
11ax	1.27 (*m*)	1.26 (*m*)	1.27 (*m*)	1.27 (*m*)	1.26 (*m*)	1.24 (*m*)
11eq	1.84 (*m*)	1.87 (*m*)	1.89 (*m*)	1.92 (*m*)	1.91 (*m*)	1.84 (*m*)
12	3.62 (*m*)	3.47 (*m*)	3.49 (*m*)	4.47 (*m*)	4.44 (*m*)	3.80 (*m*)
13	1.44 (*dd*, 13.8, 3.2)	2.16 (*dd*, 13.8, 3.2)	2.08 (*dd*, 13.8, 3.2)	2.12 (*dd*, 13.0, 3.0)	2.11 (*dd*, 13.6, 3.0)	2.78 (*dd*, 13.6, 8.0)
15ax	1.17 (*ddd*, 13.8, 13.0, 3.6)	1.76 (*ddd*, 13.8, 13.2, 3.6)	1.21 (*ddd*, 13.8, 13.3, 3.6)	3.48 (*dd*, 13.0, 3.0)	3.44 (*dd*, 13.6, 3.0)	4.55 (*d*, 7.6)
15eq	1.56 (*ddd*, 13.0, 4.0, 3.6)	1.77 (*ddd*, 13.2, 4.0, 3.6)	1.68 (*ddd*, 13.2, 4.0, 3.6)	-	-	-
16ax	1.80 (*m*)	1.70 (*m*)	1.72 (*m*)	2.02 (*m*)	1.98 (*m*)	5.63 (*dd*, 10.8, 7.6)
16eq	2.05 (*m*)	2.02 (*m*)	2.04 (*m*)	2.35 (*m*)	2.32 (*m*)	-
17	2.29 (*m*)	2.22 (*m*)	2.88 (*m*)	3.98 (*m*)	4.23 (*m*)	6.62 (*dd*, 10.8, 8.0)
18	0.91 (*s*)	0.96 (*s*)	1.06 (*s*)	0.87 (*s*)	0.85 (*s*)	0.89 (*s*)
19	0.89 (*s*)	0.93 (*s*)	0.99 (*s*)	0.99 (*s*)	0.96 (*s*)	0.98 (*s*)
21	1.45 (*s*)	1.35 (*s*)	2.24 (*s*)	-	-	-
22ax	7.86 (*d*, 5.7)	1.84 (*m*)	-	-	-	-
22eq	-	1.95(*m*)	-	-	-	-
23ax	6.06 (*d*, 5.7)	1.98 (*m*)	-	-	-	-
23eq	-	2.05(*m*)	-	-	-	-
24	-	4.93 (*m*)	-	-	-	-
28	0.79 (*s*)	0.77 (*s*)	0.78 (*s*)	0.79 (*s*)	0.77 (*s*)	0.78 (*s*)
29	0.99 (*s*)	0.97 (*s*)	0.98 (*s*)	1.01 (*s*)	0.98 (*s*)	1.03 (*s*)
30	0.84 (*s*)	0.89 (*s*)	0.91 (*s*)	1.24 (*s*)	1.19 (*s*)	1.26 (*s*)
*M*e	-	-	2.01 (*s*)	2.10 (*s*)	2.08 (*s*)	-
O *M*e	-	3.26 (*s*)	-	3.21 (*s*)	-	-

**Table 2:** ^13^C-NMR data of compounds **1**–**6** in CDCl_3_.

No.	1	2	3	4	5	6
1	38.8 (*t*)	38.8 (*t*)	39.0 (*t*)	40.1 (*t*)	39.6 (*t*)	39.6 (*t*)
2	27.1 (*t*)	27.1 (*t*)	27.2 (*t*)	34.2 (*t*)	33.9 (*t*)	33.8 (*t*)
3	78.5 (*d*)	78.4 (*d*)	81.3 (*d*)	218.8 (*s*)	218.7 (*s*)	219.2 (*s*)
4	38.9 (*s*)	38.8 (*s*)	38.9 (*s*)	47.6 (*s*)	47.2 (*s*)	47.7 (*s*)
5	55.6 (*d*)	56.0 (*d*)	56.0 (*d*)	55.5 (*d*)	55.2 (*d*)	55.8 (*d*)
6	17.8 (*t*)	17.9 (*t*)	18.0 (*t*)	19.7 (*t*)	20.2 (*t*)	20.1(*t*)
7	32.8 (*t*)	33.4 (*t*)	33.3 (*t*)	34.6 (*t*)	34.5 (*t*)	35.1 (*t*)
8	39.8 (*s*)	39.0 (*s*)	39.0 (*s*)	39.9 (*s*)	39.8 (*s*)	39.7 (*s*)
9	50.1 (*d*)	50.8 (*d*)	51.0 (*d*)	50.1 (*d*)	49.9 (*d*)	49.7 (*d*)
10	37.4 (*s*)	37.7 (*s*)	37.5 (*s*)	37.2 (*s*)	36.7 (*s*)	37.3 (*s*)
11	32.3 (*t*)	32.7 (*t*)	32.4 (*t*)	32.7 (*t*)	32.1 (*t*)	35.8 (*t*)
12	70.7 (*d*)	71.9 (*d*)	71.2 (*d*)	73.1 (*d*)	72.9 (*d*)	71.0 (*d*)
13	49.6 (*d*)	50.9 (*d*)	50.9 (*d*)	47.7 (*d*)	47.4 (*d*)	58.1 (*d*)
14	51.7 (*s*)	53.3 (*s*)	51.1 (*s*)	55.3 (*s*)	54.8 (*s*)	60.3 (*s*)
15	31.1 (*t*)	33.2 (*t*)	31.8 (*t*)	69.9 (*d*)	69.4 (*d*)	68.0 (*d*)
16	26.4 (*t*)	31.1 (*t*)	26.8 (*t*)	39.4 (*t*)	39.0 (*t*)	132.1 (*d*)
17	47.0 (*d*)	50.7 (*d*)	52.9 (*d*)	76.4 (*d*)	71.5 (*d*)	158.8 (*d*)
18	14.8 (*q*)	15.6 (*q*)	15.2 (*q*)	16.1 (*q*)	16.2 (*q*)	16.0 (*q*)
19	15.6 (*q*)	16.7 (*q*)	16.0 (*q*)	18.9 (*q*)	18.8 (*q*)	20.5 (*q*)
20	91.7 (*s*)	80.4 (*s*)	215.4 (*s*)	-	-	-
21	22.5 (*q*)	30.5 (*q*)	29.4 (*q*)	-	-	-
22	161.3 (*d*)	29.2 (*t*)	-	-	-	-
23	120.5 (*d*)	33.8 (*t*)	-	-	-	-
24	172.1 (*s*)	107.5 (*d*)	-	-	-	-
28	15.4 (*q*)	15.2 (*q*)	15.3 (*q*)	16.0 (*q*)	15.9 (*q*)	15.9 (*q*)
29	28.0 (*q*)	27.9 (*q*)	28.0 (*q*)	28.1 (*q*)	28.0 (*q*)	28.0 (*q*)
30	17.2 (*q*)	17.9 (*q*)	16.7 (*q*)	16.2 (*q*)	14.9 (*q*)	19.9 (*q*)
C=O	-	-	172.9 (*s*)	173.1 (*s*)	172.6 (*s*)	-
Me	-	-	21.7 (*q*)	22.1 (*q*)	21.3 (*q*)	-
OMe	-	55.4 (*q*)	-	58.6 (*q*)	-	-

Compound **3**, a white amorphous powder, exhibited a molecular formula of C_26_H_42_O_4_, based on the HRESIMS spectrum which showed a pseudomolecular ion at *m/z* 441.2978 [M+Na]^+^ (calcd. 441.2981). The IR absorptions at 3440 and 1705 cm^−1^ indicated the presence of OH and C=O groups, respectively. Its NMR data were similar to those of **2**, suggesting that **3** possessed a dammarane-type triterpenoid skeleton with the exception of the side chain resonances. The presence of one isolated aceto-group on C-17 was deduced by the HMBC correlation of H-17 (*δ*_H_ 2.88) with Me-21 (*δ*_C_ 29.4), and of H-13 (*δ*_H_ 2.08) and H-16 (*δ*_H_ 1.72 and 2.04) with the ketone of C-20 (*δ*_C_ 215.4) (Figure 2). Comparing the NMR data of **3** with those of compound **8**, the significant difference was that one acetyl group on C-3 in **3** took the place of the hydroxy group in **8**, which was confirmed by HMBC correlations of H**-**3 (*δ*_H_ 4.39) with C=O (*δ*_C_ 172.9) of acetyl group and of C-3 with H-1, H-5, H-28 and H-29. H-3 and H-12 were found to be *α*-oriented on the basis of ROESY cross-peaks of H-3/H-1ax and H-5, and of H-12/H-9 and H-30 (Figure 2). Thus, compound **3** was elucidated as 3*β*-*O*-acetyl-12*β*-hydroxy-23,24,25,26,27-hexanordammarane-20-one.

Compound **4** was obtained as a white amorphous solid. The HRESIMS displayed a pseudomolecular ion at *m/z* 443.2774 [M+Na]^+^ (calcd. for C_25_H_40_O_5_Na, 443.2773) consistent with a molecular formula of C_25_H_40_O_5_, corresponding to six degrees of unsaturation. The IR of **4** showed the strong broad absorption of OH (3450 cm**^−^**^1^) and C=O (1735 cm**^−^**^1^). The ^1^H-NMR spectrum exhibited the signals of six singlet Me, one OMe, and three oxygenated CH (*δ*_H_ 3.48, 3.98 and 4.47). The ^13^C-NMR and DEPT spectra showed the signals of six quaternary carbons (including one ester, one ketone, and four sp^3^ C-atoms), six CH, six CH_2_, six Me, and one MeO). The unsaturation degrees (including two C=O) indicated that the skeleton of **4** was the derivative of a four-ring triterpenoid. Based on the NMR data, **4** had a 20,21,22,23,24,25,26,27-octanordammarane triterpenoid skeleton [26]. In the HMBC spectra (Figure 2), the correlations of H-1, H-5 and H-28 with one C=O (*δ*_C_ 218.8) suggested that C-3 was oxygenated and a ketone group. In the ^1^H,^1^H-COSY spectrum, signals for a spin system of H**-**9/H-11/H**-**12**-**O**-**/ H**-**13/H**-**17**-**O**-**/H**-**16/ H**-**15**-**O were exhibited, indicating that C-12, C-15, and C-17 should be the three oxygenated CH at *δ*_C_ 73.1, 69.9, and 76.4 respectively. The HMBC correlations of H**-**12 (*δ*_H_ 4.47) with C=O (*δ*_C_ 173.1) of acetyl group, and H-17 (*δ*_H_ 3.98) with OMe (*δ*_C_ 58.6), indicated the location of AcO, MeO, and OH at C-12, C-17 and C-15, respectively (Figure 2). In the ROESY spectrum, the correlations of H**-**12/H**-**30/H**-**17, and of H**-**13/H**-**15 indicated AcO and MeO were in a *β*-orientation, while the OH at C-15 was in the *α*-orientation. Therefore, the structure of **4** was elucidated as 12*β*-*O*-acetyl-15*α*-hydoxy-17*β*-methoxy-3-oxo-20,21,22-23,24,25,26,27-octanordammanrane.

Compound **5** was obtained as a white amorphous powder, and its molecular formula was deduced as C_24_H_38_O_5_ by HR-ESI-MS and ^13^C-NMR data analysis. The IR of **5** showed the strong absorption of ester (1715 cm**^−^**^1^), ketone (1730 cm**^−^**^1^), and hydroxyl (3445 cm**^−^**^1^). The ^1^H- and ^13^C-NMR data of **5** and **4** were almost identical, indicating that **5** was also a derivative of 20,21,22,23,24,25,26,27-octanordammarane triterpenoid. Careful comparison of the NMR data of **5** with those of compound **4** showed that the only significant difference was that the singlet OMe signals in **4** was replaced by a OH group in compound **5**, which was confirmed by the upfield chemical shift of C-17 from *δ*_C_ 76.4 to 71.5. On the basis of the observation of similar NOESY data to those of **4**, the stereochemistry of **5** was expected to be the same. Accordingly, the structure of **5** was established as 12*β*-*O*-acetyl-15*α*,17*β*-dihydoxy-3-oxo-20,21,22-23,24,25,26,27-octanordammanrane.

Compound **6** was obtained as a white amorphous solid. Its molecular formula was established by HR-ESI-MS as C_22_H_34_O_3_. Strong broad absorption at 3444 cm**^−^**^1^ and 1736 cm**^−^**^1^ in the IR spectrum of **6** indicated the presence of OH and C=O, respectively. Comparison the ^1^H- and ^13^C-NMR of **6** with **5** indicated that **6** had the similar skeleton of 20,21,22,23,24,25,26,27-octanordammarane triterpenoid. The most significant difference was that the signals of CH_2_ (C-16) and oxygenated CH (C-17) in **5** was replaced by two olefinic carbon signals (*δ*_C_ 132.1 and 158.8) in **6**, indicating that the hydroxyl group at C-17 in **5** was eliminated to form the 16,17-ene in **6**. These deductions were further confirmed by the observed HMBC and ^1^H-^1^H COSY correlations. On the basis of these evidences and the spectral data, the structure of compound **6** was determined as 12*β*-*O*-acetyl-15*α*-hydoxy-3-oxo-17-en-20,21,22-23,24,25,26,27-octanordammanrane.

### 2.2. Cytotoxic Activity

All these compounds were evaluated for their *in vitro* cytotoxic potential against seven tumor cell lines by using the revised MTT method as described in the Experimental part. The results are summarized in Table 3. Octanordammarane triterpenoids **4**–**6** exhibited significant cytotoxicity against A-549 cells (lung cancer), BGC-823 cells (human gastric carcinoma), HepG2 cells (human hepatocellular carcinoma), HL-60 (human myeloid leukemia), MCF-7 cells (human breast cancer), SMMC-7721 (hepatocellular carcinoma), and W480 (colon cancer) with IC_50_values of 12.3–17.1, 11.3–18.3, and 9.4–19.1, respectively. Trinordammarane triterpenoids **2** and **7** showed moderate cytotoxic activities against all assayed seven tumor cell lines (20 μM < IC_50_ ≤ 50 μM). Hexanordammarane triterpenoids **3** and **8** possessed weak cytotoxic activities (50 μM < IC_50_ ≤ 80 μM), while no cytotoxicity was exhibited for compound **1** (IC_50_ ≥ 80 μM). The nor-dammarane triterpenoids **1**–**8** isolated from *V. mongolicum* shared the same basic skeletal structure with a wide variety of side chains. Due to their intrinsic structural variety and impressive biological activities, we suggest the viewpoint that the introduction of appropriate side chains into a planar polycyclic pharmacophore might strengthen the cytotoxic action.

**Table 3:** Cytotoxicity of compounds **1**–**8** against seven human tumor cell lines (IC_50_, μM) ^a^.

	Cell lines						
	A-549	BGC-823	HepG2	HL-60	MCF-7	SMMC-7721	W480
**1**	79.4	80.3	80.2	78.8	81.8	80.5	81.6
**2**	29.7	29.6	29.4	29.4	27.1	30.1	24.9
**3**	63.2	66.8	51.1	68.2	53.5	50.1	59.7
**4**	12.3	15.9	14.3	17.0	15.1	14.7	17.1
**5**	12.7	12.2	12.8	13.8	11.3	11.7	18.3
**6**	14.3	9.4	10.1	11.1	10.4	9.7	19.1
**7**	31.9	31.2	30.7	32.2	28.1	29.9	27.6
**8**	76.3	68.7	66.9	72.3	76.2	70.8	69.4
Doxorubicin	18.3	14.7	22.0	31.7	24.9	35.4	15.9

^a^ Doxorubicin activities are expressed as IC_50_ values in nM, and those of compounds **1**–**8** are expressed as IC_50_ values in μM. (-) IC_50_ > 100 μM.

### 2.3. Radical Scavenging Activity

The compounds **1**–**8** were tested *in vitro* for their radical scavenging activities by both the DPPH and ABTS assays. The results (Table 4) showed that compounds **1**–**8** had no activities towards the tested DPPH radical. Comparing the IC_50_ values with that shown by Trolox in the two assays, compounds **4**–**6** seem to possess higher antiradical activities than other compounds on ABTS^·+^ radicals. The differences of antiradical activities of compounds **4**–**6** between DPPH and ABTS^·+^ could be explained by the different mechanisms of the reactions for the two radicals, as has been reported for some compounds which possessed ABTS^·+^ scavenging activity, but did not exhibit DPPH scavenging activity [27].

**Table 4:** Free radical scavenging activity of compounds **1**–**8** evaluated with DPPH, ABTS^·+^ assays (IC_50_, μM).

	DPPH IC_50_	ABTS^·+^ IC_50_
**1**	276.8 ± 5	309.1 ± 6
**2**	253.1 ± 5	241.0 ± 5
**3**	244.7 ± 5	256.1 ± 5
**4**	101.3 ± 4	68.1 ± 4
**5**	99.7 ± 3	59.2 ± 3
**6**	94.1 ± 3	54.6 ± 3
**7**	261.4 ± 5	299.1 ± 5
**8**	241.0 ± 5	271.3 ± 6
Trolox	42.8 ± 1	80.1 ± 3

## 3. Experimental

### 3.1. General

Optical rotations were determined with a JASCO P2000 digital polarimeter (Tokyo, Japan). Ultraviolet (UV) and infrared (IR) spectra were obtained on JASCO V-650 and JASCO FT/IR-4100 spectrophotometers (Tokyo, Japan), respectively. NMR spectra were measured on a Bruker AM-600 spectrometer. EIMS and HREIMS (70 eV) were carried out on a Finnigan MAT 95 mass spectrometer. All solvents used were of analytical grade (Shanghai Chemical Reagents Company Ltd., Shanghai, China). Silica gel (200–300 mesh), silica gel H (Qingdao Haiyang Chemical Co. Ltd., Qingdao, China), C18 reversed-phase silica gel (150–200 mesh, Merck), and MCI gel (CHP20P, 75–150 lm, Mitsubishi Chemical Industries Ltd., Tokyo, Japan) were used for column chromatography. HPLC separation was performed on an instrument consisting of a Waters 600 controller, a Waters 600 pump, and a Waters 2487 dual λ absorbance detector, with a Prevail (250 × 10 mm i.d.) preparative column packed with C18 silica (5 μm).

### 3.2. Plant Material

The whole plants of *V. mongolicum* were collected in Hanzhong Shanxi Province, China, in April 2010. The sample was identified by one of the authors (W. Wang). A specimen (VM201004001) was deposited in the Herbarium of Shengyang Medicine College, Shengyang, China.

### 3.3. Extraction and Isolation

The dried whole plants of *V. mongolicum* (15 kg) were powdered and extracted with 80% EtOH (20 L×3) at room temperature. After removal of EtOH under reduced pressure, the aqueous brownish syrup (1 L) was suspended in H_2_O (l L) and then partitioned with chloroform (1 L×3) to afford 137 g chloroform-soluble fractions. The chloroform-soluble fraction (137 g) was further fractionated through a silica gel column, eluted with CHCl_3_–Me_2_CO (from 1:0 to 0:1), to obtain eleven fractions (1–11) according to TLC analysis. Fraction 3 (2.3 g) was applied to an ODS MPLC column and eluted with MeOH–H_2_O (20:80, 30:70, 40:60, each 500 mL) to yield four subfractions (Fr. 3–1 and 3–4). Fr. 3–2 (MeOH–H_2_O, 413 mg) was chromatographed by a Sephadex LH-20 column eluted with MeOH/CHCl_3_ (50:50), and purifed by a preparative RP-HPLC (ODS column, 250 × 20 mm) using MeOH/H_2_O (25:75) as mobile phase to afford **3** (77 mg) and **7** (63 mg). Fr. 3–3 (MeOH–H_2_O, 215 mg) was purified by a preparative RP-HPLC (ODS column, 250 × 20 mm) using MeOH/H_2_O (30:70) as mobile phase to obtain **4** (61 mg). Fraction 4 (1.5 g) was applied to an ODS MPLC column and eluted with MeOH/H_2_O (20:80, 30:70, 40:60, each 500 mL) to yield two subfractions (Fr. 4–1 and Fr. 4–2). Subfraction 4–2 (317 mg) was purified by a preparative RP-HPLC (ODS column, 250 × 20 mm) using MeOH/H_2_O (33:67) as mobile phase to obtain **2** (61 mg) and **6** (67 mg). Separation of fraction 5 (4.2 g) by silica gel column chromatography, eluted with petroleum ether–Me_2_CO (from 8:1 to 1:1), afforded six subfractions (Fr. 5–1 and Fr. 5–6). Fr. 5–2 (278 mg) was subjected to RP-18 (MeOH-H_2_O, from 2:8 to 6:4) and Sephadex LH-20 (MeOH) column chromatography to yield **1** (53 mg). Fr. 5–3 (203 mg) was repeatedly chromatographed on silica gel (chloroform:methanol, from 20:1 to 10:1) and then purifed by a Sephadex LH-20 column eluted with MeOH/H_2_O (50:50) to afford **5** (67 mg) and **8** (78 mg).

*3β,12β-Dihydroxy-25,26,27-trinordammara-22-en-24,20-olide* (**1**). White amorphous powder. 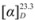 = +0.4 (*c* = 0.40, MeOH). UV (CDCl_3_) λ_max_(log *ε*): 198 (0.10) nm. IR (KBr) *ν*_max_ 3450, 2945, 2870, 1702, 1640, 1605, 1463, 1384, 1247, 1107 cm^−1^. ^1^H-NMR (CDCl_3_, 600 MHz) data see Table 1, ^13^C-NMR (CDCl_3_, 125 MHz) data see Table 2. EI-MS *m/z*: 430 ([M]^+^). HR-ESI-MS (pos.) *m/z*: 453.2984 ([M+Na]^+^, C_27_H_42_O_4_Na. calcd. 453.2981).

*3β,12β-Dihydroxy-24α-methoxy-25,26,27-trinordammara-20,24-epoxy* (**2**). White amorphous powder. 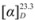 = +27.1 (*c* = 0.11, MeOH). UV (CDCl_3_) λ_max_(log *ε*): 205 (0.56) nm. IR (KBr) *ν*_max_ 3425, 2945, 2876, 1630, 1383, 1276, 1033 cm^−1^. ^1^H-NMR (CDCl_3_, 600 MHz) data see Table 1, ^13^C-NMR (CDCl_3_, 125 MHz) data see Table 2. EI-MS *m/z*: 448 ([M]^+^). HR-ESI-MS (pos.) *m/z*: 471.3454 ([M+Na]^+^, C_28_H_48_O_4_Na. calcd. 471.3450).

*3β-O-Acetyl-12β-hydroxy-23,24,25,26,27-hexanordammarane-20-one* (**3**). White amorphous powder. 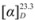 = +46.3 (*c* = 0.15, MeOH). UV (CDCl_3_) λ_max_(log *ε*): 200 (0.72) nm. IR (KBr) *ν*_max_ 3440, 2968, 2945, 2870, 1705, 1463, 1384, 1365, 1177, 1042, 1007 cm^−1^. ^1^H-NMR (CDCl_3_, 600 MHz) data see Table 1, ^13^C-NMR (CDCl_3_, 125 MHz) data see Table 2. EI-MS *m/z*: 418 ([M]^+^). HR-ESI-MS (pos.) *m/z*: 441.2978 ([M+Na]^+^, C_26_H_42_O_4_Na. calcd. 441.2981).

*12β-O-Acetyl-15α-hydroxy-17β-methoxy-3-oxo-20,21,22-23,24,25,26,27-octanordammanrane* (**4**). White amorphous powder. 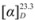 = +15.4 (*c* = 1.210, MeOH). UV (CDCl_3_) λ_max_(log *ε*): 206 (0.66) nm. IR (KBr) *ν*_max_ 3450, 1735, 1635, 1345 cm^−1^. ^1^H-NMR (CDCl_3_, 600 MHz) data see Table 1, ^13^C-NMR (CDCl_3_, 125 MHz) data see Table 2. EI-MS *m/z*: 420 ([M]^+^). HR-ESI-MS (pos.) *m/z*: 443.2774 ([M+Na]^+^, C_25_H_40_O_5_Na. calcd. 443.2773).

*12β-O-Acetyl-15α,17β-dihydroxy-3-oxo-20,21,22-23,24,25,26,27-octanordammanran* (**5**). White amorphous powder. 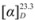 = +11.4 (*c* = 1.130, MeOH). UV (CDCl_3_) λ_max_(log *ε*): 205 (0.69) nm. IR (KBr) *ν*_max_ 3445, 1730, 1715, 1625, 1034 cm^−1^. ^1^H-NMR (CDCl_3_, 600 MHz) data see Table 1, ^13^C-NMR (CDCl_3_, 125 MHz) data see Table 2. EI-MS *m/z*: 406 ([M]^+^). HR-ESI-MS (pos.) *m/z*: calcd. 429.2613 ([M+Na]^+^, C_24_H_38_O_5_Na. calcd. 429.2617).

*12β,15α-Dihydroxy-3-oxo-17-en-20,21,22-23,24,25,26,27-octanordammanran* (**6**). White amorphous powder. 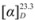 = +10.7 (*c* = 0.460, MeOH). UV (CDCl_3_) λ_max_(log *ε*): 202 (0.54) nm. IR (KBr) *ν*_max_ 3444, 1736, 1630, 1034 cm^−1^. ^1^H-NMR (CDCl_3_, 600 MHz) data see Table 1, ^13^C-NMR (CDCl_3_, 125 MHz) data see Table 2. EI-MS *m/z*: 346 ([M]^+^). HR-ESI-MS (pos.) *m/z*: 369.2405 ([M+Na]^+^, C_22_H_34_O_3_Na. calcd. 369.2406).

### 3.4. Cytotoxicity Assay *in Vitro*

The isolated compounds **1**–**8** were subjected to cytotoxic evaluation against A-549 cells (lung cancer), BGC-823 cells (human gastric carcinoma), HepG2 cells (human hepatocellular carcinoma), HL-60 (human myeloid leukemia), MCF-7 cells (human breast cancer), SMMC-7721 (hepatocellular carcinoma), and W480 (colon cancer) by employing the revised MTT method as described in the literature [28,29]. Doxorubicin was used as the positive control. All tumor cell lines were cultured on RPMI-1640 medium supplemented with 10% fetal bovine serum, 100 U mL^−1^ penicillin and 100 μg/mL streptomycin in 25 cm^2^ culture flasks at 37 °C in humidified atmosphere with 5% CO_2_. For the cytotoxicity tests, cells in exponential growth stage were harvested from culture by trypsin digestion and centrifuging at 180 ×*g* for 3 min, then resuspended in fresh medium at a cell density of 5 × 10^4^ cells per mL. The cell suspension was dispensed into a 96-well microplate at 100 μL per well, and incubated in humidified atmosphere with 5% CO_2_ at 37 °C for 24 h, and then treated with the compounds at various concentrations (0, 1, 10, 100 μM). After 48 h of treatment, 50 μL of 1 mg/mL MTT solution was added to each well, and further incubated for 4 h. The cells in each well were then solubilized with DMSO (100 μL for each well) and the optical density (OD) was recorded at 570 nm. All drug doses were tested in triplicate and the IC_50_ values were derived from the mean OD values of the triplicate tests *versus* drug concentration curves. The 50% inhibition concentration (IC_50_ value) was determined by curve fitting and was used as criteria to judge the cytotoxicity (active: IC_50_ ≤ 20 μM; moderately active: 20 μM < IC_50_ ≤ 80 μM; not active: IC_50_ > 80 μM). All cell lines were purchased from Cell Bank of Shanghai Institute of Biochemistry & Cell Biology, Chinese Academy of Sciences. Other reagents were purchased from Shanghai Sangon Biological Engineering Technology & Services Co., Ltd. (Shanghai, China).

### 3.5. Microplate Assay for Radical Scavenging Activity DPPH

Microplate DPPH assay was performed as described by [30]. Briefly, in a 96-well plate, successively sample dilutions (standard stocks of different samples 5 mM), in triplicate, received DPPH solution (40 μM in methanol) in a total volume of 0.2 mL and absorbance was measured at 550 nm with a microplate reader. Results were determined each 5 min until 60 min in order to evaluate kinetic behavior of the reaction. The percentage of remaining DPPH was calculated as follows: %DPPH_rem_ = 100 × ([DPPH]_sample_/[DPPH]_blank_). A calibrated Trolox (6-hydroxy-2,5,7,8-tetramethylchroman-2-carboxylic acid, 3.9 mM initial concentration) standard curve was also made. The percentage of remaining DPPH against the standard concentration was then plotted in an exponential regression, to obtain the amount of antioxidant necessary to decrease the initial DPPH concentration by 50% (IC_50_).

### 3.6. 2,2'-Azino-bis(3-ethylbenzothiazoline-6-sulfonic acid) (ABTS) Radical Cation Decolorization Assay

ABTS^·+^ radical scavenging activity was determined according to Re [31]. The ABTS^·+^ cation radical was produced by the reaction between 7 mM ABTS in H_2_O and 2.45 mM potassium persulfate, stored in the dark at room temperature for 16 h. Before usage, the ABTS^·+^ solution was diluted with phosphate buffer (0.05 M, pH 7.4) to get an absorbance of 0.800 ± 0.035 at 734 nm. The solution is stable for 2 days. Different concentrations of extracts or pure compounds in methanol solution were added to 1 mL of ABTS^·+^ solution. The mixture was incubated at 37 °C in the dark. After 30 min of incubation, the percentage inhibition of absorbance at 734 nm was calculated for each concentration relative to a blank absorbance (methanol). All determinations were carried out at least three times, and in triplicate. The capability to scavenge the ABTS^·+^ radical was calculated using the following equation:

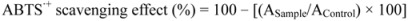

where in A_Control_ is the initial concentration of the ABTS^·+^ and A_Sample_ is absorbance of the remaining concentration of ABTS^·+^ in the presence of different compounds. Trolox was used as reference. The stock concentrations of Trolox and of different compounds tested are the same as reported in DPPH assay.

## 4. Conclusions

A chemical investigation of the 80% EtOH extract of the dried whole plants of *V. mongolicum* resulted in the isolation of six new nor-dammarane triterpenoids, 3*β,*12*β*-dihydroxy-25,26,27-trinordammara-22-en-24,20-olide (**1**), 3*β,*12*β*-dihydroxy-24*α*-methoxy-25,26,27-trinordammara-20,24-epoxy (**2**), 3*β*-*O*-acetyl-12*β*-hydroxy-23,24,25,26,27-hexanordammarane-20-one (**3**), 12*β*-*O*-acetyl-15*α*-hydroxy-17*β*-methoxy-3-oxo-20,21,22-23,24,25,26,27-octanordammanrane (**4**), 12*β*-*O*-acetyl-15*α*,17*β*-dihydroxy-3-oxo-20,21,22-23,24,25,26,27-octanordammanrane (**5**), and 12*β,*15*α*-dihydroxy- 3-oxo-17-en-20,21,22-23,24,25,26,27-octanordammanrane (**6**), together with two known compounds, 12*β*-hydroxy-3-oxo-24*α*-methoxy-25,26,27-trinordammara-20,24-epoxy (**7**) and 3*β,*12*β*-dihydroxy-23,24,25,26,27-hexanordammarane-20-one (**8**). Previous studies have indicated that significant antioxidant property of some natural products might be responsible for their antitumor property [27], suggesting that a triterpenoid possessing both antioxidant and antiproliferative activities could offer a broad spectrum of bioactivities. In our present study, all the isolated compounds were evaluated *in vitro* for their cytotoxic activities against seven tumor cell lines and radical scavenging properties. Octanordammarane tertripenoids **4**–**6** showed particular cytotoxic activities against all tested tumor cell lines, with low IC_50_ values of less than 20 μM, and exhibited radical scavenging activities against ABTS^·+^ radicals with IC_50_ values comparable with that of the standard drug Trolox.
